# Cryogenic Transport Characteristics of P-Type Gate-All-Around Silicon Nanowire MOSFETs

**DOI:** 10.3390/nano11020309

**Published:** 2021-01-26

**Authors:** Jie Gu, Qingzhu Zhang, Zhenhua Wu, Jiaxin Yao, Zhaohao Zhang, Xiaohui Zhu, Guilei Wang, Junjie Li, Yongkui Zhang, Yuwei Cai, Renren Xu, Gaobo Xu, Qiuxia Xu, Huaxiang Yin, Jun Luo, Wenwu Wang, Tianchun Ye

**Affiliations:** 1 Key Laboratory of Microelectronics Devices and Integrated Technology, Institute of Microelectronics, Chinese Academy of Sciences (CAS), Beijing 100029, China; gujie@ime.ac.cn (J.G.); zhangqingzhu@ime.ac.cn (Q.Z.); wuzhenhua@ime.ac.cn (Z.W.); yaojiaxin@ime.ac.cn (J.Y.); zhangzhaohao@ime.ac.cn (Z.Z.); zhuxiaohui@ime.ac.cn (X.Z.); wangguilei@ime.ac.cn (G.W.); lijunjie@ime.ac.cn (J.L.); zhangyongkui@ime.ac.cn (Y.Z.); caiyuwei@ime.ac.cn (Y.C.); xurenren@ime.ac.cn (R.X.); xugaobo@ime.ac.cn (G.X.); xqx@ime.ac.cn (Q.X.); luojun@ime.ac.cn (J.L.); wangwenwu@ime.ac.cn (W.W.); tcye@ime.ac.cn (T.Y.); 2 School of Electronic, Electrical and Communication Engineering, University of the Chinese Academy of Sciences, Beijing 100049, China

**Keywords:** gate-all-around, Si nanowire, cryo-CMOS, one-dimensional hole transport

## Abstract

A 16-nm-L_g_ p-type Gate-all-around (GAA) silicon nanowire (Si NW) metal oxide semiconductor field effect transistor (MOSFET) was fabricated based on the mainstream bulk fin field-effect transistor (FinFET) technology. The temperature dependence of electrical characteristics for normal MOSFET as well as the quantum transport at cryogenic has been investigated systematically. We demonstrate a good gate-control ability and body effect immunity at cryogenic for the GAA Si NW MOSFETs and observe the transport of two-fold degenerate hole sub-bands in the nanowire (110) channel direction sub-band structure experimentally. In addition, the pronounced ballistic transport characteristics were demonstrated in the GAA Si NW MOSFET. Due to the existence of spacers for the typical MOSFET, the quantum interference was also successfully achieved at lower bias.

## 1. Introduction

Nowadays, advanced silicon complementary metal oxide semiconductor (CMOS) technology is a significant platform for research of quantum computer realization. On the one hand, spin qubits based on silicon metal-oxide-semiconductor (MOS) quantum dots are a potential candidate for quantum information processing because of its material advantage [1] and compatibility with the mature CMOS fabricating technology for qubits extension and system integration. On the other hand, the cryogenic CMOS (cryo-CMOS) used for a quantum-classical interface (QCI) [2] in quantum computing system is an extraordinary research direction for a better control of power dissipation, noise and crosstalk. Si MOS quantum dot devices for spin qubits have been realized based on fully-depleted silicon on insulator (FD-SOI) technology [3,4] and sub-fin in conventional bulk fin field-effect transistor (FinFET) process [5]. The cryo-CMOS technology, based on conventional MOSFET [6,7,8], fully depleted silicon (germanium) on insulator (FD-SOI(GOI)) [9,10,11,12] and FinFET [13], has also been investigated. Some new kinds of devices [14,15,16] have now been reported for MOSFET alternatives. Gate-all-around (GAA) silicon nanowire (Si NW) or nanosheet (NS) field effect transistor (FET) is regarded as the most likely candidate to replace FinFET in the next CMOS technology nodes [17,18], and has better gate control ability for scaling down with lower power dissipation and higher integration density as well as the application for cryo-CMOS. In addition, the quantum confinement effect will be more pronounced in the small-size GAA NW devices. With the volume inversion effect in the multi-gate MOSFET [19], one-dimensional (1D) electron gas could be formed in the GAA NW channel. The carriers are only free to move along the channel direction, producing series quantized sub-band, which influences the electrical characteristics of the GAA Si NW MOSFET [20,21] and presents another potential application of nanoelectronics such as quantum information.

In this paper, we fabricated a short-channel (~16 nm) p-type GAA Si NW FET with popular RMG (replace metal gate) process based on mainstream bulk Si CMOS technology. Both the temperature dependence for the normal transistor’s characteristics and the 1D hole transport of (110) direction have been investigated systematically. Good gate controllability and body effect immunity for the cryo-CMOS are demonstrated. We also observed the two-fold degenerate sub-bands from the complicated one-dimensional (1D) hole sub-band structure and demonstrated pronounced ballistic transport characteristics in the GAA Si NW FET.

## 2. Materials and Methods

**Figure 1:**
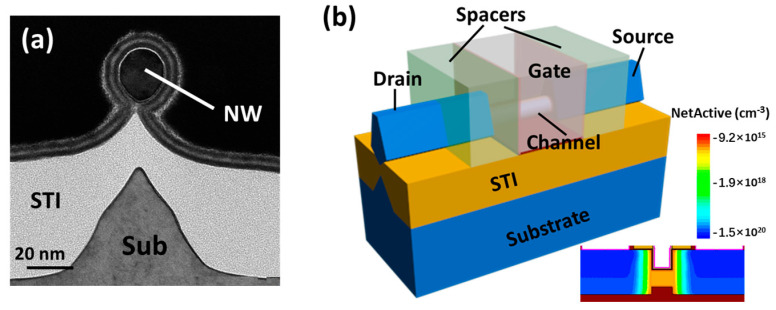
Device structure: (**a**) cross-sectional transmission electron microscopy (TEM) image perpendicular to the channel, (**b**) schematic of the Gate-all-around (GAA) silicon nanowire (Si NW) metal oxide semiconductor field effect transistor (MOSFET) with a circular nanowire channel. Inset: Simulated doping profile of the device.

The device was fabricated on 200 mm in diameter bulk silicon (100) wafers. The whole process is evolved from the bulk FinFET process and similar to the previous work [22], which is different from other Si NW synthesis with bottom up [23] or top down [24] approaches. Firstly, a spacer image transfer (SIT) technology is used for fin patterning, followed by the formation of fin with notches at both bottom sides by a special etch method [25]. The total isolated fin was formed after oxidation and the regular fabrication processes, including STI (shadow trench isolation), dummy gate formation and 40 nm thickness Si_3_N_4_ spacers were performed. The S/D (source/drain) was formed by implantation (BF^+^, dose of 2E15 and energy of 5 keV) and a rapid thermal anneal (1050 °C spike). After poly open polish (POP) process and dummy gate removal, the isolated fin channel was released using dilute HF solution and treated by H_2_ baking in the ASME 2000 plus reduced pressure chemical vapor deposition (RPCVD) chamber (20 mT, 850 °C) for 120 s for channel shrinking and rounding. Multi-layer high-k/metal gate structure was formed via atom layer deposition (ALD) process. Finally, the metal contact process were performed. Table 1 lists some important parameters of this GAA NW device. The final channel is along the (110) direction and the gate length is about 16 nm. As the transmission electron microscopy (TEM) of cross section perpendicular to the channel shown in Figure 1a, the channel is fully isolated to the substrate by a thick oxide, which is round and fully wrapped by high-k material and presents a GAA structure with 9 nm-radius nanowire channel. The equivalent oxide thickness (EOT) is about 1 nm. Figure 1b shows the schematic of this device, only part of the channel is rounded into nanowire while the other part remains big fin geometry, which reduces the S/D contact resistance and guarantees the stability of the suspended channel structure.

The electrical characteristics of this GAA Si NW device were measured in the vacuum chamber at temperatures ranging from 6 K to 300 K with Lake Shore CRX-4K system (Carson, CA, USA) and Keysight B1500 semiconductor parameter analyzer (Santa Rosa, CA, USA).

## 3. Results and Discussion

### 3.1. Cryogenic CMOS Characteristics

**Figure 2:**
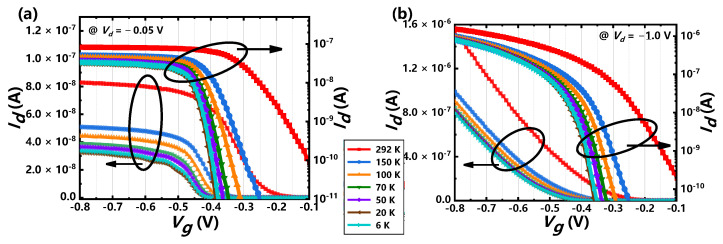
Measured linear and logarithmic *I_d_*–*V_g_* curves at different temperatures ranging from 6 K to 292 K at (**a**) *V_d_* = −0.05 V and (**b**) *V_d_* = −1 V.

**Figure 3:**
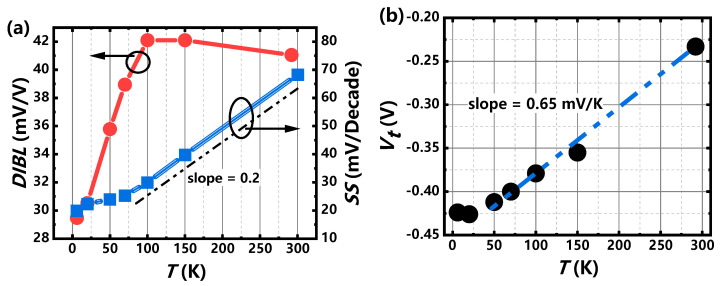
The temperature (*T*) dependence for extracted (**a**) DIBLs and SSs and (**b**) *V_t_s* of the GAA Si NW FET.

Figure 2 shows the temperature dependence of transfer curves. At the overall trend, drain current decreases as the temperature decreases and the sub-threshold characteristics become better at lower temperature. The on-state current decreases with the decrease of the temperatures, which is attribute to the suppression of thermal-activated conducting current and the temperature-induced mobility degradation for the p-type MOS transistor as temperature drops down [26]. As the metrics for the short-channel device, the DIBL (drain induced barrier lowering) effect is an electrostatic characteristic and almost insensitive to the temperature [27], which keeps stable as the temperature is above 100 K, as shown in Figure 3a. However, the effect of the drain-induced barrier lowering (DIBLs) improves obviously as the temperature drops down below 100 K, which is different from the slight degradation for SOI MOSFETs [28,29] due to the floating-body and impact ionization effects [28]. The improvement of the DIBL is mainly attributed to the increase in the surface potential bending because of freeze-out effect below 77 K [27]. On the other hand, the tunneling current that has stronger channel coupling especially in short-channel nanowire devices may contribute to the improvement of the DIBLs [10] as the thermal current is suppressed at cryogenic. The temperature dependence of subthreshold swing (SS) is perfectly matched to the theoretical function given by $\mathrm{SS}\left(T\right)=\ln10\cdot \frac{k_{B}T}{e}\approx 0.199T$ mV/Decade at *T* > 70 K, indicating the good gate controllability and body effect immunity for the short-channel GAA Si NW MOSFETs as well. The saturation of SS is observed below 50 K and the common explanation is the increase of interface state density at cryogenic [30]. As mentioned below, the gradually dominant in transport by tunneling current as temperature decrease is another factor of SS degradation for the short channel devices, since the tunneling probability is temperature independent and weaker dependent to the gate voltage compared with the subthreshold thermal current at cryogenic [31]. As the nanowire radius is small enough (~10 nm), the temperature dependence of threshold voltage for p-type MOSFET can be considered as full-depleted channel like FD-SOI [32] obtained by [33]:

(1) $$\frac{dV_{t}}{dT}=\frac{d\Phi_{F}}{dT}=8.63\times 10^{-5}\left[\ln N_{d}-38.2-\frac{3}{2}\left(1+\ln T\right)\right]\left(\mathrm{unit}:V/K\right),$$

**Figure 4:**
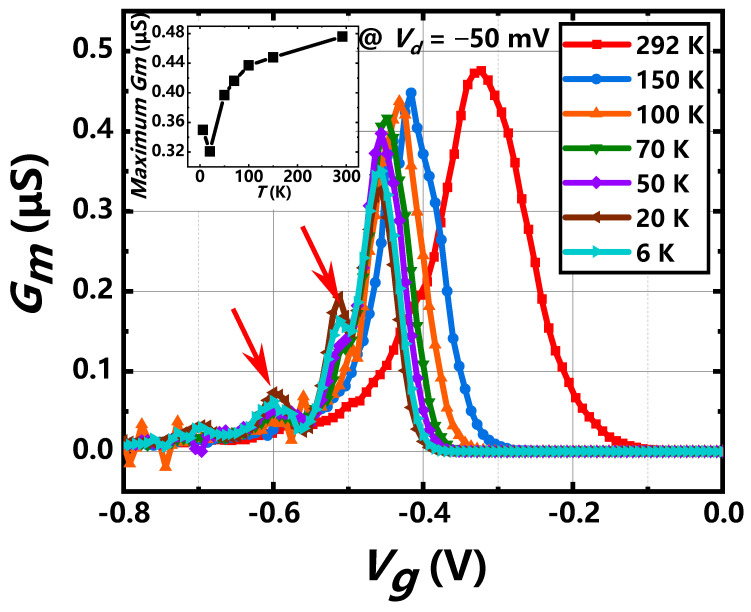
The extracted transconductance at bias of −50 mV at different temperatures ranging from 6 K to 292 K. Inset: temperature dependence of maximum *G_m_*.

The value is about 0.55–0.79 mV/K for the donor concentration *N_d_* = 1× 10^17^ cm^−2^ at the temperature ranging from 50 K to 300 K, which agrees well with the experiment result (0.65 mV/K). When the temperature is below 50 K, the incomplete ionization or freeze out effect by reducing N_d_ and innegligible quantum effect [34] at lower temperature will weaken the temperature dependent of threshold voltage, as shown in Figure 3b. Figure 4 shows the transconductance (*G_m_*) characteristics at different temperature. The position of *G_m_* peak shifts to right as the tendency of threshold voltage. The relation of peak transconductance (maximum *G_m_*) and surface mobility is given by $\mu =\frac{LG_{m}}{WC_{OX}V_{d}}$, revealing the mobility degradation as the temperature drops down at first because ionized impurity scattering is dominant at cryogenic [35]. The field effect mobility peak of this nanowire device is about 10 cm^2^/V·s, smaller than some previous publication [36], which may be attributed to a short channel and a large serious resistance by not applying epitaxy or silicide process in small S/D fin regions. As the temperature is below 20 K, the thermal energy is less than ionization energy [37], thus ionized impurity scattering is weakened, which contributes to the increase of mobility at 6 K and the enhancement of on-sate current. The other transconductance peaks (red arrows) at *T* < 50 K indicate obvious quantum transport and that will be discussed in detail, as follows.

### 3.2. Low-Dimensional Hole Transport

**Figure 5:**
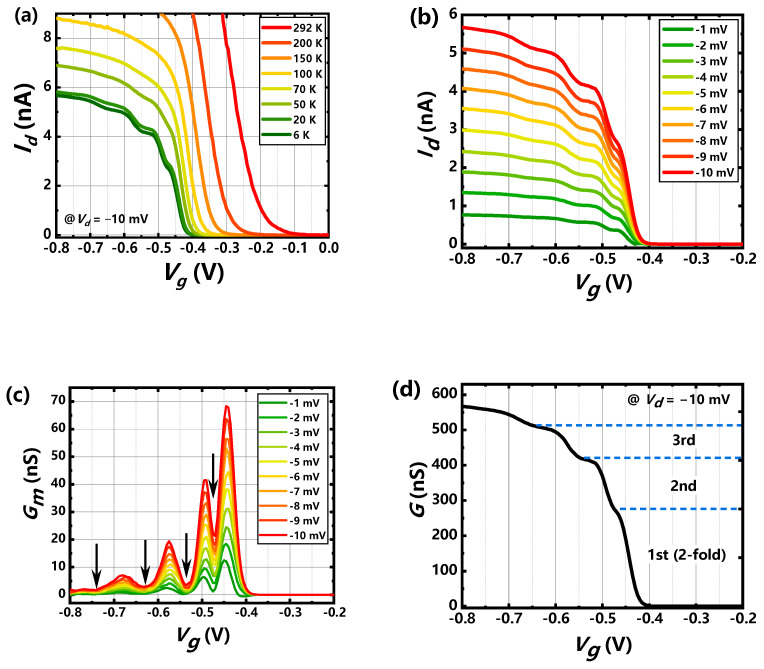
(**a**) Measured *I_d_*–*V_g_* curves at *V_d_* = −10 mV within the temperature ranging from 6 K to 292 K. (**b**) Measured *I_d_*–*V_g_* curves at different drain voltage at 6 K. (**c**) Transconductance extracted from (**b**). (**d**) The conductance as function of gate voltage for *V_d_* = −10 mV at 6 K.

The temperature dependence of drain current as the function of gate voltage at low bias was investigated. *I_d_*–*V_g_* curves exhibit step-shape current when the thermal-diffuse current is suppressed at the temperature below 50 K as shown in Figure 5a, which is the 1-D hole transport resulting from the tunneling conducting path dominated at low temperature. Several step-like currents can be observed clearly as *V_g_* exceeds the threshold voltage (~−0.42 V) from the *I_d_*–*V_g_* curves at 6 K (Figure 5b), indicating the successive hole sub-bands occupancy as *V_g_* decreases. The reduction of the *G_m_* peaks as *V_g_* reveals the quick current saturation for the carrier transport, which demonstrates the 1D quantum transport further since the 2D transport results in relative uniformly amplitude for the *G_m_* peaks [38]. Black arrows in Figure 5c mark the positions of gate voltage for *G_m_* valleys and the corresponded gate voltages are −0.47 V, −0.53 V, −0.63 V and −0.74 V, respectively. The spacing of gate voltage (Δ*V_g_*) between two adjacent *G_m_* valleys are determined to be 0.06 V, 0.1 V and 0.11 V, respectively. The experimental energy spacing of 1D sub-bands Δ*E* can be estimated by:

(2) $$\Delta E=\frac{C\cdot \Delta V_{g}\cdot \pi \hbar^{2}}{2m^{*}e}$$

where *C* is the gate capacitance per unit area and 30% degradation from the oxide capacitance according to Neophytou [39], m* is the hole effective mass. The Δ*E* can be achieved 7.3 meV, 12.2 meV and 13.4 meV, respectively. It is reasonable that the quantized step-like current can be observed below 50 K (1.7~3.1 k_B_*T*).

Figure 5d shows the conductance characteristics for *V_d_* = −10 mV at 6 K. The height for the first current step is two or more times of the second or third step, which is corresponding to the two-fold degeneracy of the lowest two-hole sub-bands for the (110) direction simulated based on k∙p method [40]. The result is different from the electron sub-bands structure in 1D nanowire that 2nd and 3rd, 4th and 5th are two-fold degeneracy, respectively [41]. The proportion of the total current for the first two-fold degenerate sub-bands is nearly 50%, adjusting well with the simulated result [40]. The relation of conductance *G* and the number of populated 1D sub-bands is given by Wharam [42]:

(3) $$G=\frac{2e^{2}}{h}\underset{i}{\sum }T_{i},$$

where *T_i_* is the transmission coefficient of the ith sub-band. The second or third current step has smaller current height, which is due to the transmission coefficient degradation because of the inter-sub-band scattering [19] as more sub-bands are populated when more negative gate voltage is applied.

**Figure 6:**
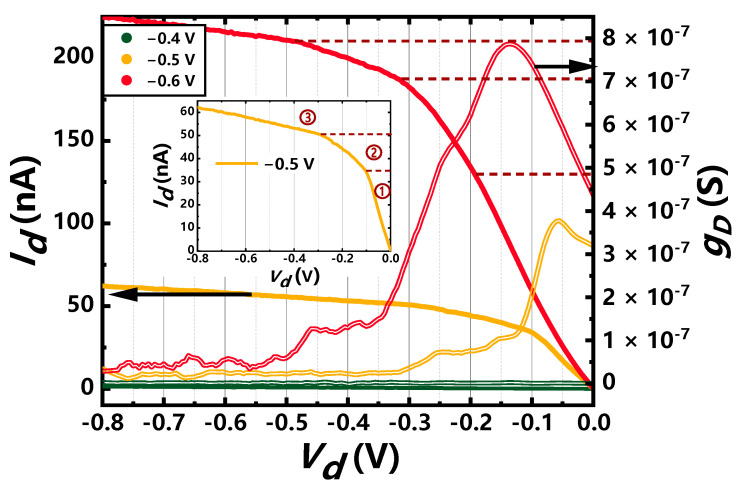
Measured *I_d_*–*V_d_* curves and channel conductance *g_D_* for different gate voltages at 6 K. Inset: the *I_d_*–*V_d_* characteristics at *V_g_* = −0.5 V.

For the fixed gate voltage, *I_d_* increases with *V_d_* at low bias given by:

(4) $$I_{d}=M\frac{2q^{2}}{h}V_{d},$$

where *M* is the current-carrying mode. The number of modes increases with gate voltage due to more sub-bands populated. Figure 6 shows the 1-D transport of *I_d_*–*V_d_* characteristics at different gate voltages for this GAA Si NW device. The channel current increases in different slopes as drain voltage decreases, reflecting step-like channel conductance. At first, the channel conductance increases as *V_d_*, which may contribute to the depression of serious inner-sub-band scattering as *V_d_* decreases. The position of abrupt change for the slope corresponding to the drain Fermi level traversing a sub-band minimum as the drain voltage becomes more negative. This phenomenon indicates the formation of great one-dimensional ballistic conducting path for this 16-nm-L_g_ channel GAA Si NW device [43].

**Figure 7:**
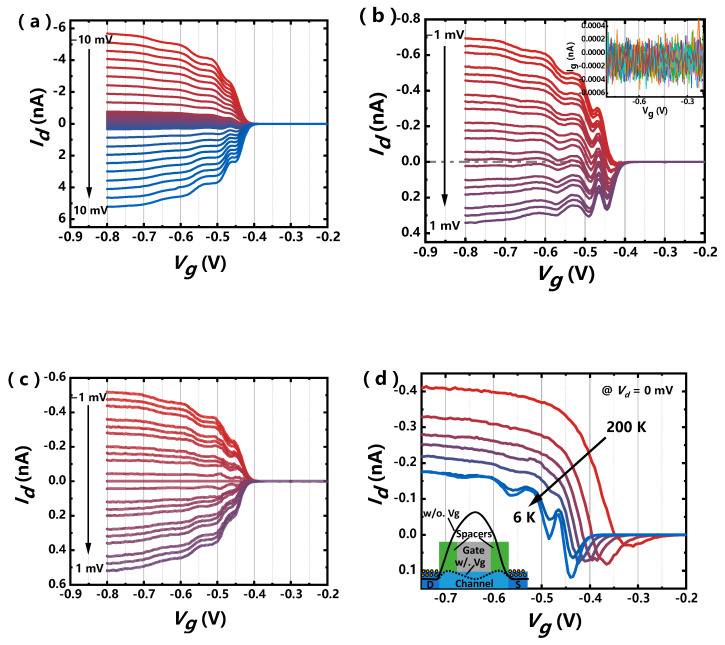
(**a**) Measured *I_d_–V_g_* curves at different *V_g_*s ranging from −10 mV to 10 mV and (**b**) from −1 mV to 1 mV at 6 K. Inset: gate current *I_g_* as function of *V_g_*. (**c**) The *I_d_–V_g_* curves at different *V_g_* by subtracting the drain zero-bias current. (**d**) Temperature dependence of zero-bias drain current with gate voltage. Inset: Electrostatic potential distribution by applying gate voltage (dotted line) or without gate voltage (solid line).

The drain current as function of gate voltage has been measured in a wider range including negative and positive drain bias as shown in Figure 7a. What is interesting is that the asymmetry for the current of negative and positive bias is observed especially at lower absolute value of bias shown in Figure 7b. The suspicion of gate leakage current is firstly ruled out from the inset of Figure 7b, and the measured gate current is about 3 orders of magnitude smaller than the drain current. The profile of *I_d_*–*V_d_* becomes symmetry if the zero-bias current is subtracted as shown in Figure 7c, which indicates that the asymmetry characteristics are almost independent from the absolute value of drain bias higher than about 100 μV. Current oscillation is observed when the thermal broadening for the carrier energy distribution is suppressed at a temperature below 50 K, which is the phenomena of quantum interference in the nanowire-conducting channel. The quantum interference in the nanoelectronics is the interference of partial charge-carrier waves between the two reflecting points [44] and also can be a signature of ballistic transport. During the fabrication of the devices, the poly gate and spacers prevent the impurities going into the channel below when S/D implantation was performed. The difference of Fermi energy between S/D and channel results in a big potential barrier between source and drain when no gate voltage is applied as the electrostatic potential distribution shown in the inset of Figure 7d. As the applied gate voltage decreases for the p-type device, the volume-inversion conducting path is formed below the threshold voltage. In addition, the big potential barrier may split into two small tunneling barriers under the spacer because of the weaker potential control for these regions as shown in Figure 7d, which contributes to the quantum interference in this kind of nanowire MOSFET. As the double tunneling barriers are not big enough, the quantum interference only occurs at bottom conducting path with lower energy level, which results in the superposition of quantum interference and 1D transport at higher bias. The height of double tunneling barriers decreases as the function of gate voltage, thus the current resonant smears out as the *V_g_* decreases. The elastic mean free path is given by Biercuk [45]:

(5) $$l=\frac{4e}{c_{g}\Delta V_{g}},$$

where the *c_g_* is the capacitance per unit length and the Δ*V_g_* is the periodicity for the gate voltage. The elastic mean free path is achieved about 9.4 nm, considering the process variation and the *V_g_*-dependent tunneling barriers, which is reasonable for the 16-nm-L_g_ nanowire device. As the gate voltage decreases, the zero-bias current shifts just like a negative bias is added. This bias offset is attributed to the asymmetric gate-induced tunneling barriers [46]. The difference of tunneling in and out rate because of the asymmetric tunneling barriers results in a finite current at zero bias at finite gate voltage [46].

## 4. Conclusions

In conclusion, 16-nm-L_g_ p-type GAA Si NW MOSFETs were fabricated based on the mainstream bulk silicon CMOS technology. The temperature dependence of electrical characteristics for the normal MOSFET has been systematically investigated, which demonstrates a good gate-control ability and body effect immunity as well as the contribution of tunneling current at cryogenic. Moreover, a great 1D hole transport was characterized in the same device. The two-fold degeneracy in hole sub-bands at (110) channel direction was experimentally observed from the complicated nanowire hole sub-bands structure. These results demonstrate the potential of cryo-CMOS as well as a great physical platform of quantum device for GAA Si NW devices compatible with the next generation CMOS technology.

## Figures and Tables

**Table 1:** Some important parameters of the GAA NW device.

Gate Length	EOT	NW Diameter	Channel Concentration	S/D Concentration
16 nm	1 nm	18 nm	1 × 10^17^ cm^−3^	1 × 10^20^ cm^−3^

## Data Availability

The data presented in this study are available on request from the corresponding authors.
